# Effects of light on quantum phases and topological properties of two-dimensional Metal-organic frameworks

**DOI:** 10.1038/srep41644

**Published:** 2017-01-30

**Authors:** Yunhua Wang, Yulan Liu, Biao Wang

**Affiliations:** 1 Sino-French Institute of Nuclear Engineering and Technology, Sun Yat-sen University, Zhuhai 519082, China; 2 State Key Laboratory of Optoelectronic Materials and Technologies, Sun Yat-sen University, Guangzhou 510275, China; 3 School of Engineering, Sun Yat-sen University, Guangzhou 510275, China

## Abstract

Periodically driven nontrivial quantum states open another door to engineer topological phases in solid systems by light. Here we show, based on the Floquet-Bloch theory, that the on-resonant linearly and circularly polarized infrared light brings in the exotic Floquet quantum spin Hall state and half-metal in two-dimensional Metal-organic frameworks (2D MOFs) because of the unbroken and broken time-reversal symmetry, respectively. We also observe that the off-resonant light triggers topological quantum phase transitions and induces semimetals with pseudospin-1 Dirac-Weyl fermions via the photon-dressed topological band structures of 2D MOFs. This work paves a way to design light-controlled spintronics and optoelectronics based on 2D MOFs.

Once discovered in materials, quantum states, with extra degrees of freedom, unconventional conical bands or nontrivial topological features, could yield entirely new physics and device paradigms in nanoelectronics and information technology. The first example is the half-metallic state with 100% spin polarization near the Fermi energy, where the spin degree of freedom can be used as information carriers in spintronics^1^. The second example is the semimetallic state in graphene with linear electronic band dispersion associated with the Dirac physics^2^. As a counterpart of the electronic spin, the extra valley degree of freedom used as information carriers in graphene, silicene or monolayer transition metal dichalcogenides could lead to the exotic valleytronics^3^,^4^. The third example is the topological insulators (TIs), where fully spin-polarized currents carried by the robust conducting edge or surface states inside the insulating bulk gap allow TIs for applications in spintronics and quantum computation^5^,^6^. In addition, more exotic quantum states have also been explored recently, such as Weyl semimetals^7^,^8^,^9^, axion insulators and three-dimensional Dirac semimetals^10^,^11^. Besides searching for materials with these exotic quantum states, engineering these states in condensed matter or nanostructures by external fields also has aroused tremendous attention during the past few years.

Via photon-dressed band structures and properties in Floquet-Bloch picture, light-matter interaction not only offers novel experimental and theoretical platforms for engineering Floquet topological insulating phases^12^,^13^,^14^,^15^,^16^,^17^,^18^,^19^,^20^,^21^,^22^,^23^,^24^,^25^,^26^,^27^,^28^,^29^,^30^ and semimetallic phases^31^,^32^,^33^,^34^,^35^,^36^ in solid systems, but also sparks the same interest in photonic crystals and optical lattices^37^,^38^,^39^,^40^,^41^,^42^. These Floquet quantum states not only display similar behaviours as their counterparts in static system but also exhibit additional features, which require the extension of the classifications^13^,^43^,^44^,^45^,^46^,^47^ and are directly manifested by their unique nonequilibrium transport properties^16^,^17^,^18^,^48^,^49^,^50^,^51^,^52^,^53^. In general, the Floquet-Bloch theory, which describes the interaction of light with Bloch states in solids, can be divided into two classes in view of two distinct physical mechanisms. The first is based on the zeroth static Floquet Hamiltonian in the off-resonant regime, where the driving frequency ω is larger than the bandwidth Λ of the undriven system. In this case, the real absorption and emission for a photon with frequency *ω* between the uncoupled Floquet sidebands are unlikely, but the virtual photon absorption and emission^17^ can incorporate with the Bloch electrons and renormalize the electronic structures. The second is governed by the truncated Floquet Hamiltonian in the on-resonant regime (*ω* < Λ), where the overlapped Floquet sidebands and the photon resonances are responsible for these exotic Floquet quantum states^15^,^20^,^24^,^43^.

In this work, we focus on the coherent interaction of light with the recently discovered two-dimensional Metal-organic frameworks (2D MOFs)^54^,^55^,^56^,^57^, in both of the off-resonant and on-resonant Floquet-Bloch pictures. Owing to the numerous combinations of different metal ions and organic ligands, 2D MOFs have various chemical structures and versatile physical and chemical functionalities^58^, such as topological electronic properties^59^,^60^,^61^,^62^,^63^,^64^,^65^,^66^, Dirac semimetals^67^, half-metallicity^68^ and chemiresistive response^69^. The dominant nearest-neighbor hopping (0.01 eV < *t*_1_ < 0.1 eV)^59^,^60^,^61^,^62^,^63^,^64^,^65^,^66^,^67^,^68^ in 2D MOFs is much less than that in graphene (*t*_1_ ~ 2.8 eV)^2^, and hence only needs the coupling light with a lower frequency (ω < 1.2 × 10^14^ Hz) in infrared, which is experimentally accessible^19^. Consequently, the infrared sensitivity opens a door to engineer quantum states in 2D MOFs by light. Herein, we report the effects of infrared light on the quantum phases and topological properties of M_3_C_12_S_12_ (M is a metal ion, such as Ni, Cu, Pt, Au and others)^58^, a kind of 2D MOFs with kagome lattice (Fig. 1a and b), within the framework of the Floquet-Bloch physics. It is shown that photoinduced quantum phases in M_3_C_12_S_12_ can be attributed to the Floquet-Peierls (FP) substitutions, which allow the effective lattices to be engineered through the renormalized hoppings as well as the spin-orbit couplings (SOC) and permit the Floquet quantum phases to be customized by the photon-dressed band structures and topological properties. Under the off-resonant light irradiation, the nonzero FP substitutions maintain the kagome lattice but with modified strengths, which can reverse three energy bands of M_3_C_12_S_12_ with different spin chern numbers and hence trigger a topological quantum phase transition. Single zero FP substitution transforms the kagome lattice into the topologically equivalent Lieb lattice, which supports the semimetals with the Pseudospin-1 Dirac-Weyl fermions. Under the on-resonant light irradiation, the circularly polarized light with frequency (Λ/2 < *ω* < Λ) induces robust Floquet half-metal by virtue of the broken time-reversal symmetry, but the linearly polarized light with lower frequency (*t*_1_ < *ω* < Λ/2) brings in the exotic Floquet quantum spin Hall state with the gapless helical edge states protected by the time-reversal symmetry. These results demonstrate that Dirac semimetals, Floquet half-metal and Floquet topological insulating states can be engineered in the same 2D MOFs by tuning the driving parameters (frequency, amplitudes and polarization) of light, and therefore open a new way to design light-controlled spintronics and optoelectronics based on 2D MOFs.

## Results

### The zeroth static Floquet Hamiltonian

**Figure 1:**
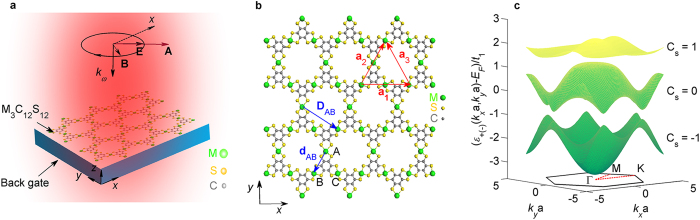
Photon-dressed topological band structures of M_3_C_12_S_12_. (**a**) Schematic of light-irradiated M_3_C_12_S_12_ on a back gate controlling the Fermi energy *E*_*F*_. Infrared light with the incident wave vector *k*_*ω*_ travels along the negative z axis (perpendicular to the M_3_C_12_S_12_ plane) and induces the time-dependent vector potential **A**(*t*) = (*A*_*x*_sin(*ω*t), *A*_*y*_sin(*ω*t + *ϕ*), 0) with the frequency *ω*. (**b**) 2D kagome lattice of M_3_C_12_S_12_. Here, **a**_1_ = (1, 0)a, 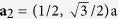, and **a**_3_ = **a**_2_−**a**_1_ are the lattice vectors with the lattice constant a, **d**_*ij*_ (*i, j* = A, B or C, *i.e.*, the sublattices) is the nearest-neighbor vector, and **D**_*ij*_ is the next-nearest-neighbor vector. (**c**) The photon-dressed topological band structures of M_3_C_12_S_12_ with a group of spin chern numbers (−1, 0, 1) from bottom up, for *A*_*x*_a = 1.5, *A*_*y*_a = 1.5, *ϕ* = 0, and *λ*_1_ = 0.14*t*_1_.

First, let us consider the effects of light on M_3_C_12_S_12_ (Fig. 1a) in the off-resonant regime (*ω* > Λ). In this case, the Floquet sidebands are uncoupled, and the photon-dressed band structures can be captured by the zeroth static Floquet Hamiltonian (see Methods in details)


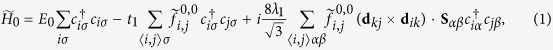


where 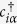 and *c*_*iα*_ are the creation/annihilation operators for an electron with the spin *α* on site **r**_*i*_, **S** is the spin Pauli matrix, and **d**_*kj*_ is the nearest-neighbor (denoted by 〈*i, j*〉) vector pointing from site **r**_*j*_ to **r**_*k*_ (see Fig. 1b). The constant on-site energy *E*_0_ just shifts the whole energy spectrum, and hence is usually set to the zero energy^2^. The nearest-neighbor hopping energy *t*_1_ and the intrinsic SOC strength *λ*_1_ are modified by these FP substitutions


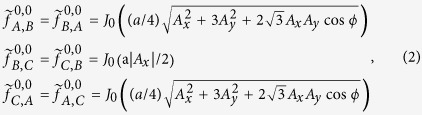


where *A*_*x*_ and *A*_*y*_ are the amplitudes, *ϕ* is the phase difference reflecting the polarization of light, and *J*_0_(*x*) is the zeroth Bessel function of the first kind. The zeroth static Floquet Hamiltonian in equation (1) shows that the effects of the off-resonant light on the electronic properties of M_3_C_12_S_12_ are decided by the FP substitutions, which allow us to design effective lattices by tuning the driving parameters (*A*_*x*_, *A*_*y*_ and *ϕ*) of light: (1) If 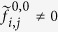, the hopping lattice of the irradiated M_3_C_12_S_12_ remains the kagome lattice but with modified real hoppings that keep the time-reversal invariant, and hence M_3_C_12_S_12_ maintains the topological insulating phases (Fig. 1c) because of the SOC; (2) If single FP substitution is zero, the hopping framework will become topologically equivalent to the Lieb lattice, which directly supports the semimetals with the pseudospin-1 Dirac-Weyl fermions near the Dirac points^70^,^71^,^72^,^73^,^74^; (3) If two or three FP substitutions are zero, the driven hopping lattice will be correspondingly equivalent to a quantum wire or some discrete lattice points such that 2D MOFs are always semimetals because of the touched conduction and valence bands with zero band gap.

### Phase diagram and topological quantum phase transitions

**Figure 2:**
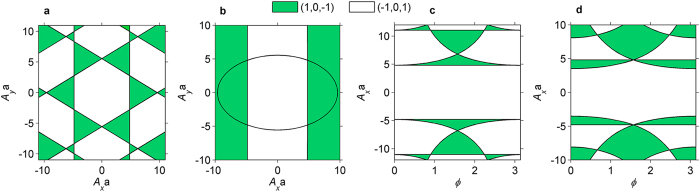
Phase diagram of light-irradiated M_3_C_12_S_12_. (**a**) Phase diagram in the (*A*_*x*_, *A*_*y*_) plane for linearly polarized light (*ϕ* = 0 or π). (**b**) Phase diagram in the (*A*_*x*_, *A*_*y*_) plane for circularly polarized light (*ϕ* = *π*/2). (**c,d**) Phase diagrams in the (*ϕ, A*_*x*_) plane for elliptically polarized light with 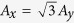 and *A*_*x*_ = *A*_*y*_, respectively.

**Figure 3:**
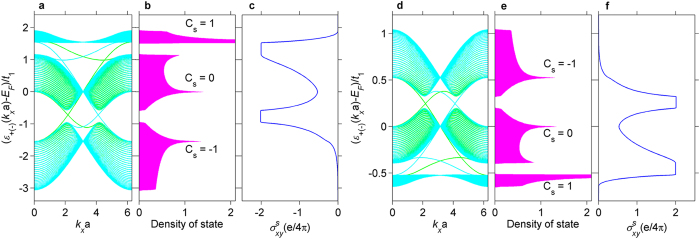
Edge state spectra of light-irradiated M_3_C_12_S_12_ on a cylindrical geometry. For topological insulating phases (−1, 0, 1) with light parameters (*A*_*x*_a = 2, *A*_*y*_a = 2, and *ϕ* = *π*/2) and (1, 0, −1) with light parameters (*A*_*x*_a = 6, *A*_*y*_a = 6, and *ϕ* = *π*/2), respectively: The spin-up (green) and spin-down (cyan) edge state spectra in (**a,d**), the density of state in (**b,e**) and the spin Hall conductance in (**c,f**).

We consider the phase diagrams of M_3_C_12_S_12_ subjected to the light irradiation with linear, circular and elliptical (arbitrary) polarizations, respectively. In Fig. 2 we construct the phase diagrams in the (*A*_*x*_, *A*_*y*_) plane for linearly and circularly polarized light and in the (*ϕ, A*_*x*_) plane for elliptically polarized light. In the case of the off-resonance that keeps the time-reversal symmetry, the topological insulating phases can still be characterized by a group of spin Chern numbers (C_s_)^47^,^75^,^76^ or Kane-Mele invariants^77^ for the three distinct energy bands. From these phase diagrams, we can see that the off-resonant light induces two different topological insulating phases with the spin Chern numbers (−1, 0, 1) and (1, 0, −1), respectively. The topological phase transition occurs at the boundaries between the two different topological insulating phases, where the band gap is closed and the semimetal appears. In addition, the phase distributions are symmetrical owing to the symmetries of 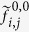 with respect to the amplitudes *A*_*x*_ and *A*_*y*_ as well as the phase difference *ϕ* (see equation (2)). On the other hand, the edge state is a powerful tool to reveal the topological features of energy bands and search for the topological phase transitions, because of the bulk-edge correspondence. For TIs, fully spin-polarized gapless helical edge states protected by time-reversal symmetry are directly responsible for the spin Hall conductance (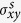). Therefore, the change of the spin Hall conductance can provide a signature of topological phase transitions. We calculate the edge state spectrum, the density of state and the spin Hall conductance for the two distinct TIs with *λ*_1_ = 0.14*t*_1_ (Fig. 3), on a cylindrical geometry, *i.e.*, a 34-unit-cell open boundary condition in the *y* direction and a periodic boundary condition in the *x* direction. As expected, the three bands of M_3_C_12_S_12_ for the two different topological insulating phases are reversed, and a pair of robust spin-filtered gapless states inside each bulk gap leads to the contrary values of the quantized spin Hall conductance. As a result, the off-resonant light triggers the topological quantum phase transition between the two phases (−1, 0, 1) and (1, 0, −1).

### Pseudospin-1 Dirac-Weyl fermions and flat band

**Figure 4:**
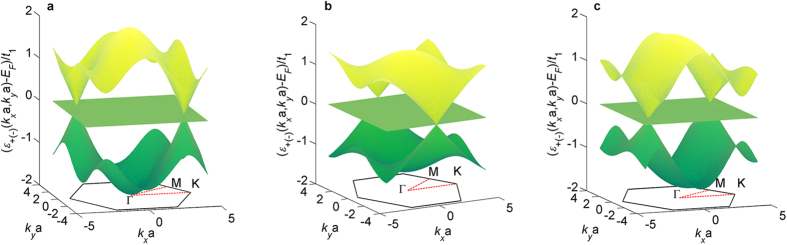
Light-induced semimetals with pseudospin-1 Dirac-Weyl fermions in M_3_C_12_S_12_. (**a**) The photon-dressed band structure for case (*i*): 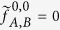, 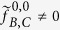, and 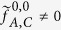 with light parameters *A*_*x*_a = 2, *A*_*y*_a = 4.4, and *ϕ* = 0. (**b**) The photon-dressed band structure for case (*ii*): 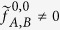, 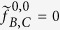, and 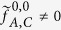 with light parameters *A*_*x*_a = 4.81, *A*_*y*_a = 2, and *ϕ* = *π*/2. (**c**) The photon-dressed band structure for case (*iii*): 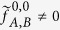, 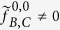, and 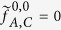 with light parameters *A*_*x*_a = 2, *A*_*y*_a = 6.71, and *ϕ* = 0.

In the above section, we concentrate on the topological insulating phases and phase transitions. Here we focus on the semimetals induced by the three cases: (*i*) 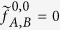, 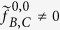, and 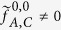, (*ii*) 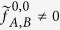, 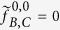, and 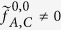, (*iii*) 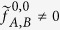, 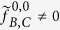, and 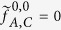. We elucidate the analytical expressions of photon-dressed energies *ε*_±_(**k**) and Hamiltonians 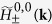 for the semimetal phases in the three cases (see Supplementary Note 1). The obtained energy band structures of the semimetals for the three cases with *λ*_1_ = 0.2*t*_1_ are shown in Fig. 4. Distinct from the light-induced extra Dirac cones at the surface of a topological insulator^21^ or in graphene^23^, two conical bands touch at the Dirac points, and an additional flat band exists. This band structure is the typical energy spectrum of the pseudospin-1 Dirac-Weyl fermions^70^,^71^,^72^,^73^,^74^. In this case, the spin degeneracy of the energy band is not lifted because of the time-reversal symmetry for the off-resonant light and the space-inversion symmetry of the light-engineered Lieb lattice in M_3_C_12_S_12_ (Supplementary Note 1). The obtained effective Hamiltonians near the Dirac points (*D*_*x*_, *D*_*y*_) in the three cases can be rewritten as the general form of the pseudospin-1 Dirac-Weyl fermions: 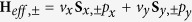 (see Supplementary Note 2) with the anisotropic group velocities *v*_*x*_ and *v*_*y*_, a new wave vector **p** = (*p*_*x*_, *p*_*y*_, 0) and the pseudospin vectors **S**_±_ = (**S**_*x*_,_±,_ **S**_*y*_,_±,_ **S***z*,_±_), which satisfies 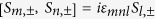 with the Levi-Civita symbol *ε*_*mnl*_. The expressions of these quantities are given in Supplementary Table 1, where **p** = **Aq** with **A,** as a corresponding deformation operator similar to the manipulation of the in-plain strain^78^. Recently, searching for flat band has been particularly interesting, because the dispersionless state in the presence of Coulomb interactions can induce correlated quantum states, including ferromagnetism, superconductivity and fractional quantum Hall effect^79^,^80^,^81^,^82^. Recent experiments have shown that the localized flat band occurs in a photonic Lieb lattice that consists of an array of optical waveguides^83^,^84^. However, the flat band in real material has not been observed, since few 2D materials have the desired Lieb lattice. Here we predict the localized flat band that results from the destructive interference of electron hoppings rather than disorders or impurities by means of the light-engineered Lieb lattice in 2D MOFs.

### Truncated Floquet Hamiltonian, Floquet half-metal and Floquet quantum spin Hall insulator

**Figure 5:**
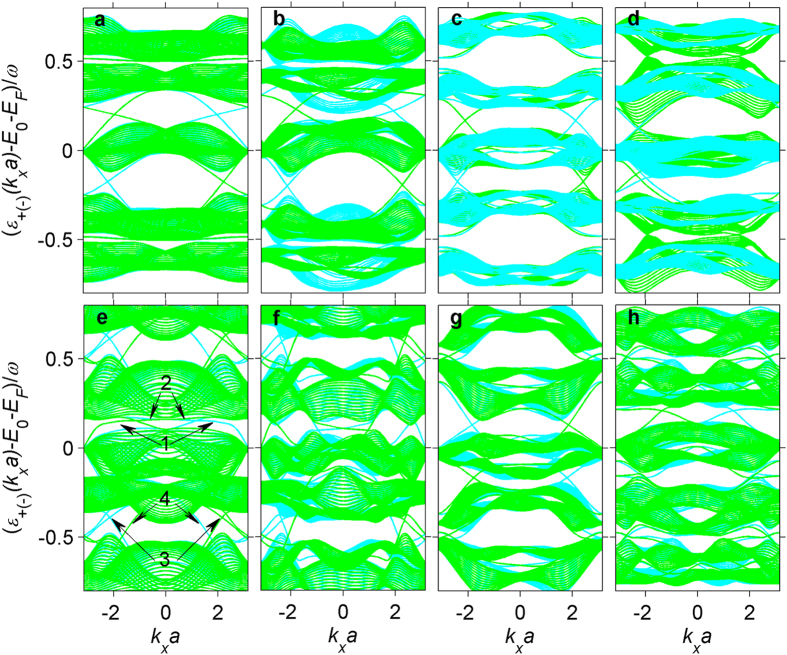
Quasienergy spectra of truncated Floquet Hamiltonian for light-irradiated M_3_C_12_S_12_ on a cylindrical geometry. Quasienergy spectrum for linearly (**a**) and circularly (**b**) polarized light with *A*_*x*_a = 2.5, *A*_*y*_a = 2.5, and *ω* = 3*t*_1_. Quasienergy spectrum for linearly (**c**) and circularly (**d**) polarized light with *A*_*x*_a = 6.5, *A*_*y*_a = 6.5, and *ω* = 2*t*_1_. Quasienergy spectrum for linearly polarized light: (**e**) *A*_*x*_a = 1.5, *A*_*y*_a = 1.5, and *ω* = 2.4*t*_1_; (**f**) *A*_*x*_a = 0.5, *A*_*y*_a = 2.5, and *ω* = 2*t*_1_; (**g**) *A*_*x*_a = 6, *A*_*y*_a = 0, and *ω* = 2*t*_1_; and (**h**) *A*_*x*_a = 8, *A*_*y*_a = 2, and *ω* = 2*t*_1_. Here E_0_ = 0, the spin-up and spin-down quasienergy spectra are denoted by green and cyan lines, respectively, and the electron densities for the edge states 1, 2, 3 and 4 in Fig. 5e are shown in Supplementary Fig. 2.

In the above sections, the zeroth static Floquet Hamiltonian predicts the light-induced topological phase transitions and the pseudospin-1 Dirac-Weyl fermions in 2D MOFs, but with the decrease of driving frequency (*t*_1_ < *ω* < Λ) the Floquet sidebands overlap such that the resonant absorptions or emissions of photons cannot be captured by the zeroth Floquet Hamiltonian. In this case, the Floquet Hamiltonian with infinite dimensions (−∞ < *m, n* < + ∞) should be considered in principle. However, the Floquet indexes *m* and *n* can be truncated to a finite order *M*, because the FP substitution 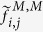 vanishes with the Bessel function of the first kind *J*_*n >M*_(*x*) ~ 0 (where *M* is a positive integer greater than *x*)^85^ and makes the Floquet states *ϕ*_*n*,*m*_ decay rapidly if *m* and *n* are beyond the finite range *M* in frequency domain. The truncated Floquet Hamiltonian of 2D MOFs, with 3*M* × 3*M* dimensions in the Sambe space^86^, includes the resonant processes of few and multiple photons beyond the weak intensity limit, which only takes the single-photon absorption or emission into account. Based on the truncated Floquet Hamiltonian, we calculate the quasienergy spectra (Fig. 5) of M_3_C_12_S_12_ irradiated by the on-resonant light with *λ*_1_ = 0.2*t*_1_, on the same cylindrical geometry as in Fig. 3. Unlike the harmonic driving of electric field always with the time-reversal symmetry^87^, the on-resonant light driving keeps the time-reversal invariant for the linear polarization, *i.e., ε*_+_ (*k*_*x*_*a*)=*ε*_*−*_(−*k*_*x*_*a*)^88^ but breaks the time-reversal symmetry for the circular polarization, *i.e., ε*_+_ (*k*_*x*_*a*) ≠ *ε*_*−*_(−*k*_*x*_*a*)^14^,^15^,^16^,^17^,^18^,^19^,^25^. As a consequence, the on-resonant linearly polarized light only induces the dynamical gap near ±*ω*/2, and has few influences on the spin-polarized gapless helical edge states inside the two native bulk gaps of the undriven M_3_C_12_S_12_ (Fig. 5a and c). However, the on-resonant circularly polarized light induces a dynamical gap for one spin but metals for the other spin (Fig. 5b and d), which results in the 100% spin-polarization, *i.e.*, the typical half-metallicity, owing to the broken time-reversal symmetry. The driven half-metal near the boundary (±*ω*/2) of the quasienergy Brillouin zone is without an analog in the undriven system, and hence is here named as Floquet half-metal. In addition, no matter the driving intensity is strong or weak, the Floquet half-metal can remain inside a limited frequency range (Λ/2 < *ω* < Λ) before the dynamical gap for both spins closes. Therefore, the Floquet half-metal is robust against the deviations of the optical parameters and the SOC intensity (see Supplementary Fig. 1). On the other hand, when the driving frequency of the linearly polarized light decreases further and becomes lower than Λ/2, some new gapless helical edge states protected by the time-reversal symmetry appear in the dynamical gap, which first closes and then reopens (Fig. 5e–h). In this case, M_3_C_12_S_12_ is converted into the Floquet quantum spin Hall insulator^47^,^87^ if the Fermi level is inside the dynamical gap. These new and initial gapless helical edge states exhibit well localizations at the two open boundaries of the ribbon, and can coexist inside the same system with few couplings because of the big energy difference between each other (Supplementary Fig. 2).

## Discussion

In this section, we first comment on the experimental feasibility to probe the predicted pseudospin-1 Dirac-Weyl fermions and the light-induced novel topological quantum phases in 2D MOFs. The Angle-resolved photoemission spectroscopy (ARPES) is a useful tool to map the electronic band dispersions of topological materials^5^,^6^, by virtue of its important information on the kinetic energy and the emission angle of emitted photoelectrons. Recently, ARPES not only has been applied to acquire the Floquet-Bloch bands of TIs (Bi_2_Se_3_) irradiated by the monochromatic infra light with tunable intensity, frequency and polarization^19^, but also has been used to distinguish the Floquet-Bloch states from the Volkov states, *i.e.*, the photon-dressed free-electron states near the surface of TIs^89^. Besides, both of the occupied and unoccupied energy bands near and far away the Fermi level can be resolved by the one-photon and two-photon ARPES^90^. Other methods are also proposed to check the Floquet-Bloch states in Floquet TIs. For instance, the mean orbital magnetization, as a result of the Floquet topological edge currents, has been suggested as a hallmark signature of the light-induced Floquet topological quantum states^91^. In addition, various 2D MOFs have been synthesized in recent experiments by the bottom-up method^54^,^55^,^56^,^57^, and the Fermi level of the single-atom-layer material can be well controlled by the back gate^92^. Therefore, we believe that the predicted light-induced pseudospin-1 Dirac-Weyl energy spectrum and the Floquet-Bloch topological band dispersions in 2D MOFs can be probed by a combination of the ARPES and the gate-controllable Fermi level.

Next, we summarize our results and present an outlook for future investigations. We explore the effects of light on the quantum phases and the topological properties of 2D MOFs with kagome lattice (M_3_C_12_S_12_) within the framework of the off-resonant and on-resonant Floquet-Bloch physics. It is shown that unusual Floquet quantum states can be engineered in the same 2D MOFs by virtue of highly tunable parameters of light. For instance, the claimed nontrivial Floquet quantum spin Hall states in the driven 2D lattice system^47^ as well as the cold atom system^87^ and the theoretically predictable^70^,^71^,^72^,^73^,^74^ and experimentally observable^83^,^84^ pseudospin-1 Dirac-Weyl fermions with flat bands in the photonic Lieb lattice are realized in the light-irradiated 2D MOFs. Moreover, we also observe that a new Floquet half-metallic state can be engineered in 2D MOFs by the on-resonant circularly polarized light that breaks the time-reversal symmetry. These results not only facilitate the developments of Floquet-Bloch physics in condensed matter, but also open a new path towards light-controlled spintronics and optoelectronics based on 2D MOFs. On the other hand, as a starting point, light-irradiated 2D MOFs also raise many interesting subjects, which deserve further explorations in future. Firstly, in the presence of interactions, periodically driven system exhibits novel Floquet many-body states^28^,^93^,^94^,^95^,^96^,^97^, such as the fractional Chern insulator states^28^, which generically support the fractional quantum Hall states^79^,^80^,^81^,^82^. Our results has demonstrated that the expected topological flat band with a large flatness ratio, which is a crucial condition for the occurrence of the fractional quantum Hall effect^79^,^80^,^81^,^82^, can be engineered by the off-resonant light in 2D MOFs (see Figs 3a,d and 4). In addition, the strong electronic correlations can be introduced by choosing the different combinations of metal ions and organic ligands. Therefore, the light-irradiated 2D MOFs may offer theoretical and experimental platforms to realize the fractional Chern insulator states in real materials. Secondly, a spatial modulation of light allows for remarkably tuning the Floquet topological properties of semiconductor quantum wells^22^ and the surface states of three-dimensional topological insulators^98^. However, how about the situation in 2D crystal materials, *i.e.*, the 2D MOFs with the kagome lattice under the spatially nonuniform irradiations? Thirdly, the temperature-dependent 2D topological phases have been characterized by the Uhlmann geometric phase^99^,^100^. It remains unclear, however, how to characterize the Floquet topological phases at finite temperature in 2D MOFs and other materials. Finally, after the above question of the temperature-dependent Floquet topological phases is addressed, and the experimental measurements on the Floquet topological quantum states in light-irradiated 2D materials are completed, Further experiments are required to explore the coupling mechanism between the Floquet TIs and the external reservoirs^52^, the electron occupations of the nonequilibrium Floquet states^101^,^102^ and the dc Hall conductance of the driven Floquet TIs^48^.

## Methods

### Tight-binding (TB) model on the kagome lattice in 2D MOFs

The equivalent single-orbital TB Hamiltonian of the kagome lattice^79^,^103^, describing the interactions between the *π* orbitals of the ligands and the *d* orbitals of the metal ions in 2D MOFs^58^,^59^,^60^,^61^,^62^,^63^,^64^,^65^,^66^, should in principle involve the nearest-neighbor and next-nearest-neighbor interactions, and can be written as


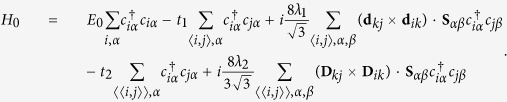


Here the first term is the on-site energy. The second and third terms are the nearest-neighbor (denoted by 〈*i, j*〉) hopping and intrinsic SOC with energy parameters *t*_1_ and *λ*_1_, respectively. The last two terms are the next-nearest-neighbor (denoted by 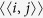) hopping and intrinsic SOC with energy parameters *t*_2_ and *λ*_2_, respectively. **S** is the spin Pauli matrix. **d**_*kj*_ and **D**_*kj*_ are the nearest-neighbor and next-nearest-neighbor vectors pointing from site **r**_*j*_ to **r**_*k*_, respectively (Fig. 1b). The factors 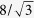 and 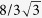 correspondingly ensure the vectors **d**_*kj*_ and **D**_*kj*_ normalized to the unite vectors, similar to that in graphene^75^ or silicene^26^. In fact, owing to *t*_1_ ≫ *t*_2_ and *λ*_1_ ≫ *λ*_2_^59^,^60^,^61^,^62^,^63^,^64^,^65^,^66^, the nearest-neighbor interactions are the main components, and hence the quite weak next-nearest-neighbor interactions are usually not considered in 2D MOFs^59^,^60^,^61^.

### Floquet-Bloch theory in the light-irradiated M_3_C_12_S_12_

In the presence of monochromatic infrared light with its spatially slowly varying electromagnetic potential **A**(*t*) = (*A*_*x*_sin(*ω*t), *A*_*y*_sin(*ω*t + *ϕ*), 0) (Fig. 1a), the time-dependent TB Hamiltonian as a result of the Peierls substitution has the following form:


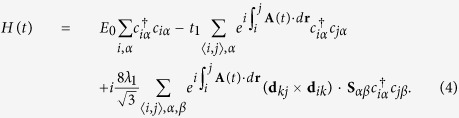


We further perform the following Fourier transforms^20^


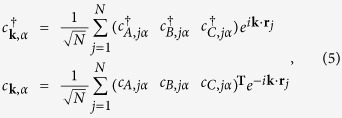


where *N* is the number of sites with periodic boundary conditions, **T** denotes the transpose operation, **k** is the wave vector defined in the Brillouin zone, and *A, B* and *C* are the three sublattices of a unit cell in kagome lattice (Fig. 1b). Then we can rewrite the time-dependent Hamiltonian in momentum space as


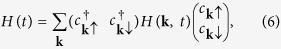


where 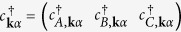 and 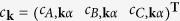 are the creation/annihilation operators for an electron with the spin *α* (↑ and ↓ denote spin up and down, respectively) in momentum space. Due to [**S**_*z*_, *H*(*t*)] = 0, the 6 × 6 matrix *H* (**k**, t) can be decoupled into two 3 × 3 spin-dependent Hamiltonians:


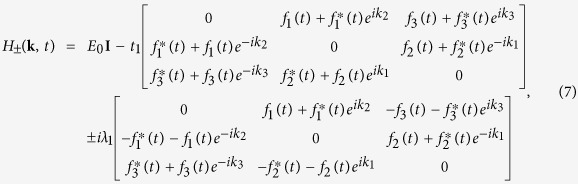


where *k*_*i*_ = **k · a**_*i*_, **I** is the 3 × 3 unite matrix, +(−) refers to spin-up (spin-down), and the time-dependent Peierls substitutions are


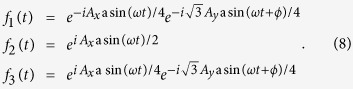


Employing the Floquet theorem, we can write Floquet-Bloch ansatz as


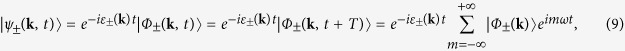


with the period *T*, the spin-dependent quasienergy *ε*_±_(**k**) and the Floquet-Bloch states 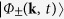. The Floquet operator 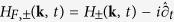 yields the time-independent Floquet energy eigenvalue equation in the Sambe space as


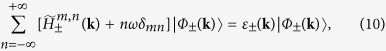


where the time-independent Hamiltonian 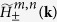 with the Floquet indexes (*m, n*) includes the emissions or absorptions of *q* photons (*q* = *m* − *n*) and takes the form:


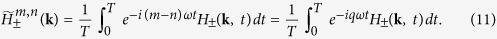


Substituting equations (6) and (7) into equation (10), we find that 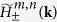 takes the same forms as the undriven static Hamiltonian but with new hopping integrals and SOC strengths modified by the time-averaged Peierls (Floquet-Peierls) substitutions:


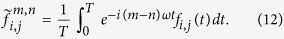


### Spin chern number and spin Hall conductance

The absence of Rashba SOC in M_3_C_12_S_12_ conserves the spin rotational symmetry, and hence the spin-dependent chern number 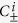 of the energy band *i* can be directly calculated using the Kubo formula


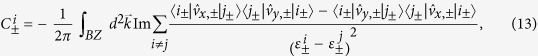


where +(−) refers to spin-up (spin-down), 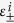 (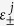) is the spin-dependent eigenvalue of the energy band *i (j*), and 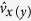 is the velocity operator. The chern number of band *i* is 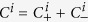 and the spin chern number of band *i* is 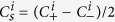. From the spin Chern number, we can further write the spin Hall conductance as 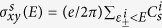.

## Additional Information

**How to cite this article**: Wang, Y. *et al*. Effects of light on quantum phases and topological properties of two-dimensional Metal-organic frameworks. *Sci. Rep.* **7**, 41644; doi: 10.1038/srep41644 (2017).

**Publisher's note:** Springer Nature remains neutral with regard to jurisdictional claims in published maps and institutional affiliations.

## Supplementary Material

Supplementary Information
