# Supplementary material for: Effects of light on quantum phases and topological properties of two-dimensional Metal-organic frameworks
Source: Sci Rep. 2017 Jan 30;7:41644. doi: 10.1038/srep41644 (PMC5278405; doi:10.1038/srep41644)
Supplement: Supplementary Information [file srep41644-s1.pdf]

Effects of light on quantum phases and topological properties of two-dimensional Metal-organic frameworks

–Supplementary Information–

Yunhua Wang[1,3,*], Yulan Liu[2] & Biao Wang[1,3,*]

[1]Sino-French Institute of Nuclear Engineering and Technology, Sun Yat-sen University, Zhuhai 519082, China.

[2]School of Engineering, Sun Yat-sen University, Guangzhou 510275, China.

[3]State Key Laboratory of Optoelectronic Materials and Technologies, Sun Yat-sen University, Guangzhou 510275, China.

Correspondence and requests for materials should be addressed to Y. H. W. (email: wangyh49@mail.sysu.edu.cn) or to B. W. (email: wangbiao@mail.sysu.edu.cn).

I. Supplementary Figures

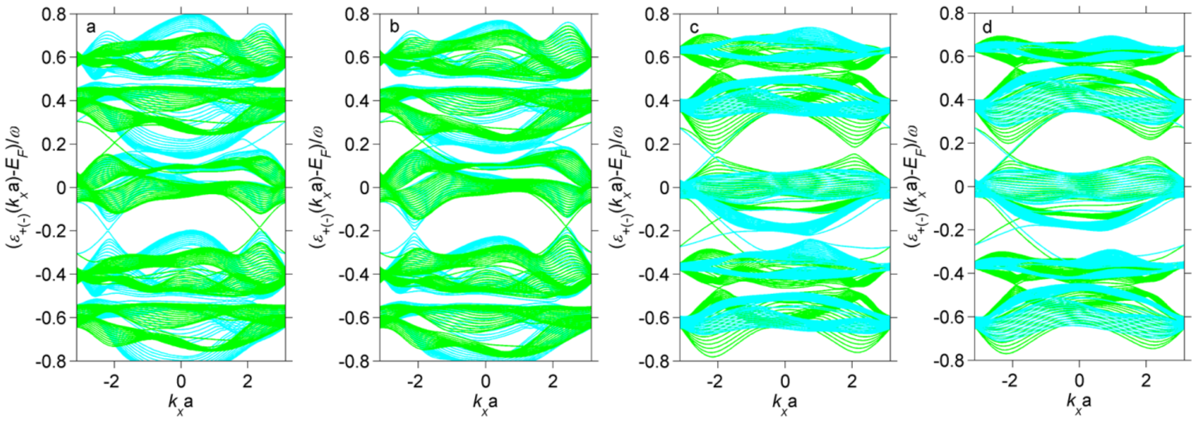

Supplementary Figure 1. Floquet half-metal induced by the on-resonant circularly polarized light in $M_3C_{12}S_{12}$. Quasienergy spectra with A_xa = 3, A_ya = 3, and $\omega = 2.4t_1$, for (**a**) $\lambda_1 = 0.2t_1$, and (**b**) $\lambda_1 = 0.1t_1$. Quasienergy spectrum with A_xa = 7, A_ya = 7, and $\omega = 2t_1$, for (**c**) $\lambda_1 = 0.2t_1$, and (**d**) $\lambda_1 = 0.1t_1$. Here the spin-up and spin-down quasienergy spectra are denoted by green and cyan lines, respectively.

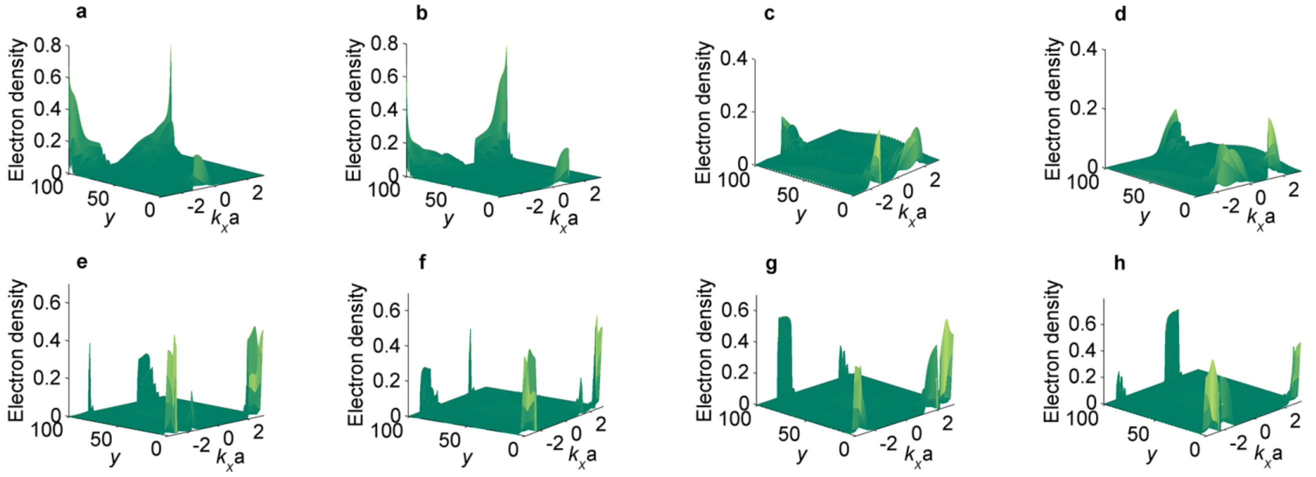

Supplementary Figure 2. Spin-resolved electron density for edge states in $M_3C_{12}S_{12}$ irradiated by the on-resonant linearly polarized light. (**a**,**c**,**e**,**g**) Spin-up electron density for edge states 1, 2, 3 and 4 in Fig. 5e, respectively. (**b**,**d**,**f**,**h**) Spin-down electron density for edge states 1, 2, 3 and 4 in Fig. 5e, respectively.

II. Supplementary Notes

Supplementary Note 1: Spin-dependent energies $\varepsilon_\pm(\mathbf{k})$ and Hamiltonian $\tilde{\mathcal{H}}_{\pm}^{0,0}(\mathbf{k})$ of the semimetal states in the three cases.

For case (*i*): $\tilde{f}_{A,B}^{0,0}=0$, $\tilde{f}_{B,C}^{0,0}\neq 0$, and $\tilde{f}_{A,C}^{0,0}\neq 0$, the spin-dependent Hamiltonian in momentum space takes the form:

$$\tilde{\mathcal{H}}_{\pm}^{0,0}(\mathbf{k})=\begin{bmatrix} 0 & 0 & \tilde{f}_{A,C}^{0,0}(t_1\mp i\lambda_1)\left(1+e^{i\left(-k_x+\sqrt{3}k_y\right)a/2}\right) \\ 0 & 0 & \tilde{f}_{B,C}^{0,0}(t_1\pm i\lambda_1)\left(1+e^{-ik_xa}\right) \\ \tilde{f}_{A,C}^{0,0}(t_1\pm i\lambda_1)\left(1+e^{-i\left(-k_x+\sqrt{3}k_y\right)a/2}\right) & \tilde{f}_{B,C}^{0,0}(t_1\mp i\lambda_1)\left(1+e^{ik_xa}\right) & 0 \end{bmatrix}, \quad (1)$$

which results in the spin-degenerated energies

$$\begin{aligned} \varepsilon_{1,\pm}(\mathbf{k}) &= -\sqrt{2\left(t_1^2+\lambda_1^2\right)}\sqrt{\left(\tilde{f}_{B,C}^{0,0}\right)^2\left(1+\cos(k_xa)\right)+\left(\tilde{f}_{C,A}^{0,0}\right)^2\left[1+\cos\left(\left(k_x-\sqrt{3}k_y\right)a/2\right)\right]} \\ \varepsilon_{2,\pm}(\mathbf{k}) &= 0 \\ \varepsilon_{3,\pm}(\mathbf{k}) &= \sqrt{2\left(t_1^2+\lambda_1^2\right)}\sqrt{\left(\tilde{f}_{B,C}^{0,0}\right)^2\left(1+\cos(k_xa)\right)+\left(\tilde{f}_{C,A}^{0,0}\right)^2\left[1+\cos\left(\left(k_x-\sqrt{3}k_y\right)a/2\right)\right]} \end{aligned} \quad . \quad (2)$$

For case (*ii*): $\tilde{f}_{A,B}^{0,0}\neq 0$, $\tilde{f}_{B,C}^{0,0}=0$, and $\tilde{f}_{A,C}^{0,0}\neq 0$, the spin-dependent Hamiltonian in momentum space takes the form:

$$\tilde{\mathcal{H}}_{\pm}^{0,0}(\mathbf{k})=\begin{bmatrix} 0 & \tilde{f}_{A,B}^{0,0}(t_1\pm i\lambda_1)\left(1+e^{i\left(k_x+\sqrt{3}k_y\right)a/2}\right) & \tilde{f}_{A,C}^{0,0}(t_1\mp i\lambda_1)\left(1+e^{i\left(-k_x+\sqrt{3}k_y\right)a/2}\right) \\ \tilde{f}_{A,B}^{0,0}(t_1\mp i\lambda_1)\left(1+e^{-i\left(k_x+\sqrt{3}k_y\right)a/2}\right) & 0 & 0 \\ \tilde{f}_{A,C}^{0,0}(t_1\pm i\lambda_1)\left(1+e^{-i\left(-k_x+\sqrt{3}k_y\right)a/2}\right) & 0 & 0 \end{bmatrix}, \tag{3}$$

which results in the spin-degenerated energies

$$\begin{aligned} \varepsilon_{1,\pm}(\mathbf{k}) &= -\sqrt{2\left(t_1^2+\lambda_1^2\right)}\sqrt{\left(\tilde{f}_{A,B}^{0,0}\right)^2\left(1+\cos\left(\left(k_xa+\sqrt{3}k_ya\right)/2\right)\right)+\left(\tilde{f}_{A,C}^{0,0}\right)^2\left(1+\cos\left(\left(k_xa-\sqrt{3}k_ya\right)/2\right)\right)} \\ \varepsilon_{2,\pm}(\mathbf{k}) &= 0 \\ \varepsilon_{3,\pm}(\mathbf{k}) &= \sqrt{2\left(t_1^2+\lambda_1^2\right)}\sqrt{\left(\tilde{f}_{A,B}^{0,0}\right)^2\left(1+\cos\left(\left(k_xa+\sqrt{3}k_ya\right)/2\right)\right)+\left(\tilde{f}_{A,C}^{0,0}\right)^2\left(1+\cos\left(\left(k_xa-\sqrt{3}k_ya\right)/2\right)\right)} \end{aligned}. \tag{4}$$

For case (*iii*): $\tilde{f}_{A,B}^{0,0}\neq 0$, $\tilde{f}_{B,C}^{0,0}\neq 0$, and $\tilde{f}_{A,C}^{0,0}=0$, the spin-dependent Hamiltonian in momentum space takes the form:

$$\tilde{\mathcal{H}}_{\pm}^{0,0}(\mathbf{k})=\begin{bmatrix} 0 & \tilde{f}_{A,B}^{0,0}(t_1\pm i\lambda_1)\left(1+e^{i\left(k_x+\sqrt{3}k_y\right)a/2}\right) & 0 \\ \tilde{f}_{A,B}^{0,0}(t_1\mp i\lambda_1)\left(1+e^{-i\left(k_x+\sqrt{3}k_y\right)a/2}\right) & 0 & \tilde{f}_{B,C}^{0,0}(t_1\pm i\lambda_1)\left(1+e^{-ik_xa}\right) \\ 0 & \tilde{f}_{B,C}^{0,0}(t_1\mp i\lambda_1)\left(1+e^{ik_xa}\right) & 0 \end{bmatrix}, \tag{5}$$

which results in the spin-degenerated energies

$$\begin{aligned} \varepsilon_{1,\pm}(\mathbf{k}) &= -\sqrt{2\left(t_1^2+\lambda_1^2\right)}\sqrt{\left(\tilde{f}_{B,C}^{0,0}\right)^2\left(1+\cos(k_xa)\right)+\left(\tilde{f}_{A,B}^{0,0}\right)^2\left[1+\cos\left(\left(k_x+\sqrt{3}k_y\right)a/2\right)\right]} \\ \varepsilon_{2,\pm}(\mathbf{k}) &= 0 \\ \varepsilon_{3,\pm}(\mathbf{k}) &= \sqrt{2\left(t_1^2+\lambda_1^2\right)}\sqrt{\left(\tilde{f}_{B,C}^{0,0}\right)^2\left(1+\cos(k_xa)\right)+\left(\tilde{f}_{A,B}^{0,0}\right)^2\left[1+\cos\left(\left(k_x+\sqrt{3}k_y\right)a/2\right)\right]} \end{aligned}. \tag{6}$$

Supplementary Note 2: Effective Hamiltonians of pseudospin-1 Dirac-Weyl fermions near the Dirac points in the three cases.

By introducing a new wave vector $\mathbf{q}=\left(q_x,q_y\right)$ measured from the Dirac points $\mathbf{D}=\left(D_x,D_y\right)$, we can rewrite the TB Hamiltonians in supplementary equations (1), (3) and (5) with $\mathbf{k}\to\mathbf{D}+\mathbf{q}$, and then expand these TB Hamiltonians to the first order of $\mathbf{q}$ and to the second order as the neglected impulses, as follows:

For case (i): $\tilde{f}_{A,B}^{0,0}=0$, $\tilde{f}_{B,C}^{0,0}\neq 0$, and $\tilde{f}_{A,C}^{0,0}\neq 0$, the Dirac points are at $\mathbf{D}=\left(\pm\pi,\mp\pi/\sqrt{3}\right)$, and the spin-dependent effective Hamiltonian reads

$$\mathcal{H}_{eff,\pm}(\mathbf{k})=\begin{bmatrix} 0 & 0 & a\tilde{f}_{A,C}^{0,0}\left(it_1\pm\lambda_1\right)\dfrac{q_x-\sqrt{3}q_y}{2} \\ 0 & 0 & a\tilde{f}_{B,C}^{0,0}\left(it_1\mp\lambda_1\right)q_x \\ a\tilde{f}_{A,C}^{0,0}\left(-it_1\pm\lambda_1\right)\dfrac{q_x-\sqrt{3}q_y}{2} & a\tilde{f}_{B,C}^{0,0}\left(-it_1\mp\lambda_1\right)q_x & 0 \end{bmatrix}. \quad (7)$$

For case (ii): $\tilde{f}_{A,B}^{0,0}\neq 0$, $\tilde{f}_{B,C}^{0,0}=0$, and $\tilde{f}_{A,C}^{0,0}\neq 0$, the Dirac points are at $\mathbf{D}=\left(0,\pm 2\pi/\sqrt{3}\right)$, and the spin-dependent effective Hamiltonian reads

$$\mathcal{H}_{eff,\pm}(\mathbf{k})=\begin{bmatrix} 0 & a\tilde{f}_{A,B}^{0,0}\left(-it_1\pm\lambda_1\right)\dfrac{q_x+\sqrt{3}q_y}{2} & a\tilde{f}_{A,C}^{0,0}\left(it_1\pm\lambda_1\right)\dfrac{q_x-\sqrt{3}q_y}{2} \\ a\tilde{f}_{A,B}^{0,0}\left(it_1\pm\lambda_1\right)\dfrac{q_x+\sqrt{3}q_y}{2} & 0 & 0 \\ a\tilde{f}_{A,C}^{0,0}\left(-it_1\pm\lambda_1\right)\dfrac{q_x-\sqrt{3}q_y}{2} & 0 & 0 \end{bmatrix}. \quad (8)$$

For case (iii): $\tilde{f}_{A,B}^{0,0}\neq 0$, $\tilde{f}_{B,C}^{0,0}\neq 0$, and $\tilde{f}_{A,C}^{0,0}=0$, the Dirac points are at $\mathbf{D}=\left(\pm\pi,\pm\pi/\sqrt{3}\right)$, and the spin-dependent effective Hamiltonian reads

$$\mathcal{H}_{eff,\pm}(\mathbf{k})=\begin{bmatrix} 0 & a\tilde{f}_{A,B}^{0,0}\left(-it_1\pm\lambda_1\right)\dfrac{q_x+\sqrt{3}q_y}{2} & 0 \\ a\tilde{f}_{A,B}^{0,0}\left(it_1\pm\lambda_1\right)\dfrac{q_x+\sqrt{3}q_y}{2} & 0 & a\tilde{f}_{B,C}^{0,0}\left(it_1\mp\lambda_1\right)q_x \\ 0 & a\tilde{f}_{B,C}^{0,0}\left(-it_1\mp\lambda_1\right)q_x & 0 \end{bmatrix}. \quad (9)$$

Note that we have set the on-site energy E_0 at the zero energy in Supplementary equations (7), (8) and (9).

III. Supplementary Table

	Case (*i*)	Case (*ii*)	Case (*iii*)
	$\tilde{f}_{A,B}^{0,0}=0,\ \tilde{f}_{B,C}^{0,0}\neq 0,\ \tilde{f}_{A,C}^{0,0}\neq 0.$	$\tilde{f}_{A,B}^{0,0}\neq 0,\ \tilde{f}_{B,C}^{0,0}=0,\ \tilde{f}_{A,C}^{0,0}\neq 0.$	$\tilde{f}_{A,B}^{0,0}\neq 0,\ \tilde{f}_{B,C}^{0,0}\neq 0,\ \tilde{f}_{A,C}^{0,0}=0.$
$\mathbf{S}_{x,\pm}$	$\begin{pmatrix} 0 & 0 & \pm e^{\pm i\theta} \\ 0 & 0 & 0 \\ \pm e^{\mp i\theta} & 0 & 0 \end{pmatrix}$	$\begin{pmatrix} 0 & \pm e^{\mp i\theta} & 0 \\ \pm e^{\pm i\theta} & 0 & 0 \\ 0 & 0 & 0 \end{pmatrix}$	$\begin{pmatrix} 0 & \pm e^{\mp i\theta} & 0 \\ \pm e^{\pm i\theta} & 0 & 0 \\ 0 & 0 & 0 \end{pmatrix}$
$\mathbf{S}_{y,\pm}$	$\begin{pmatrix} 0 & 0 & 0 \\ 0 & 0 & \mp e^{\mp i\theta} \\ 0 & \mp e^{\pm i\theta} & 0 \end{pmatrix}$	$\begin{pmatrix} 0 & 0 & \pm e^{\pm i\theta} \\ 0 & 0 & 0 \\ \pm e^{\mp i\theta} & 0 & 0 \end{pmatrix}$	$\begin{pmatrix} 0 & 0 & 0 \\ 0 & 0 & \mp e^{\mp i\theta} \\ 0 & \mp e^{\pm i\theta} & 0 \end{pmatrix}$
$\mathbf{S}_{z,\pm}$	$\begin{pmatrix} 0 & ie^{\pm 2i\theta} & 0 \\ -ie^{\mp 2i\theta} & 0 & 0 \\ 0 & 0 & 0 \end{pmatrix}$	$\begin{pmatrix} 0 & 0 & 0 \\ 0 & 0 & -ie^{\pm 2i\theta} \\ 0 & ie^{\mp 2i\theta} & 0 \end{pmatrix}$	$\begin{pmatrix} 0 & 0 & ie^{\mp 2i\theta} \\ 0 & 0 & 0 \\ -ie^{\pm 2i\theta} & 0 & 0 \end{pmatrix}$
p_x	$\left(q_x-\sqrt{3}q_y\right)/2$	$\left(q_x+\sqrt{3}q_y\right)/2$	$\left(q_x+\sqrt{3}q_y\right)/2$
p_y	q_x	$\left(q_x-\sqrt{3}q_y\right)/2$	q_x
v_x	$a\tilde{f}_{A,C}^{0,0}\sqrt{t_1^2+\lambda_1^2}$	$a\tilde{f}_{A,B}^{0,0}\sqrt{t_1^2+\lambda_1^2}$	$a\tilde{f}_{A,B}^{0,0}\sqrt{t_1^2+\lambda_1^2}$
v_y	$a\tilde{f}_{B,C}^{0,0}\sqrt{t_1^2+\lambda_1^2}$	$a\tilde{f}_{A,C}^{0,0}\sqrt{t_1^2+\lambda_1^2}$	$a\tilde{f}_{B,C}^{0,0}\sqrt{t_1^2+\lambda_1^2}$
(D_x, D_y)	$\left(\pm\pi, \mp\pi/\sqrt{3}\right)$	$\left(0, \pm 2\pi/\sqrt{3}\right)$	$\left(\pm\pi, \pm\pi/\sqrt{3}\right)$

Supplementary Table 1. The expressions of the general effective Hamiltonians $\mathcal{H}_{eff,\pm}=v_xS_{x,\pm}p_x+v_yS_{y,\pm}p_y$ for the pseudospin-1 Dirac-Weyl fermions in the light-irradiated 2D MOFs for the three cases. Here $\mathbf{S}_\pm=\left(\mathbf{S}_{x,\pm},\mathbf{S}_{y,\pm},\mathbf{S}_{z,\pm}\right)$ is the pseudospin vector, $\mathbf{p}=\left(p_x,\ p_y,0\right)$ is the wave vector, v_x and v_y are the group velocities along the *x* and *y* directions, respectively, (D_x, D_y) is the position of Dirac points, and the defined parameter θ satisfies $\cos\theta=\lambda_1/\sqrt{t_1^2+\lambda_1^2}$ and $\sin\theta=t_1/\sqrt{t_1^2+\lambda_1^2}$.
